# Neurofeedback for functional neurological disorders: future directions for research and clinical practice

**DOI:** 10.1098/rstb.2024.0468

**Published:** 2026-09-17

**Authors:** Daniela Di Basilio, Alexander J. Casson, Anthony Jones

**Affiliations:** ^1^ Faculty of Health and Medicine, Lancaster University, Lancaster, UK; ^2^ Department of Electrical and Electronic Engineering, University of Manchester, Manchester, UK; ^3^ University of Manchester, Manchester, UK

**Keywords:** functional neurological disorder (FND), brain plasticity, neurofeedback, digital health, personalized medicine

## Abstract

Functional neurological disorder (FND) is increasingly conceptualized as a disorder of network-level dysfunction involving altered salience processing, impaired motor intention–execution coupling and disrupted self-agency. Despite advances in multi-disciplinary rehabilitation, a proportion of individuals experience persistent or fluctuating symptoms, highlighting the need for mechanistically informed adjunctive interventions. Neurofeedback, a closed-loop technique enabling individuals to modulate neural activity in real time, offers a theoretically grounded approach to targeting dysfunctional circuits implicated in FND. This opinion article synthesizes current neuroscientific models of FND with evidence from neurofeedback research in motor, affective and pain-related conditions. We examine modality–target considerations (e.g. electroencephalography versus functional magnetic resonance imaging), learning mechanisms including operant and associative paradigms and the relevance of network-based interventions for heterogeneous FND phenotypes. Given the limited direct evidence in FND, we critically appraise methodological variability in existing neurofeedback applications and outline pragmatic steps towards greater standardization, including transparent reporting of neural targets, feedback modality, training dose and transfer assessment. We also discuss translational challenges, including integration within established physiotherapy frameworks, patient-centred personalization and digitally enabled delivery models. While empirical evidence remains preliminary, neurofeedback represents a biologically informed and potentially scalable intervention that warrants systematic investigation within multi-disciplinary FND care pathways.

This article is part of the discussion meeting issue ‘Digital healthcare for the management of functional neurological disorders’.

## 1. Background

### (a) Functional neurological disorder: definition and clinical considerations on the need to expand our ‘clinical toolkit’

Functional neurological disorder (FND) presents with neurological symptoms that are inconsistent with structural disease, such as limb weakness, seizures and sensory disturbances [1,2]. FNDs are now recognized as brain-based disorders with disruptions in motor control, emotion regulation and self-agency [3]. However, treatment remains complex, and many individuals experience persistent symptoms despite engagement in various forms of physical and psychological support [4]. Evidence suggests that multi-disciplinary approaches, particularly those integrating physiotherapy with psychological interventions like cognitive-behavioural therapy (CBT), may offer the greatest clinical benefit for many individuals with FND. More specifically, physiotherapy has demonstrated particular effectiveness in addressing functional motor symptoms, focusing on retraining movement patterns and reducing maladaptive attention to symptoms. CBT, instead, has been shown to be helpful in addressing typical psychological features associated with FND, such as irrational and dysfunctional beliefs (e.g. ‘it's all in my head, my illness is not real’) [5], as well as emotional responses associated with symptom persistence [6–8].

Despite these advances, there remains a substantial subgroup of patients who do not respond well to conventional therapies. As a result, there is growing interest in expanding the therapeutic toolkit to include interventions such as hypnotherapy, which has shown promise in modulating symptom expression in certain FND presentations [9–11], and non-invasive brain stimulation techniques like transcranial magnetic stimulation, which may target underlying neurophysiological abnormalities [12]. These novel approaches highlight the importance of personalized, brain-based treatments that align with emerging models of FND.

Recent developments in neurotechnology have enabled novel interventions aimed at directly modifying brain circuits. Neurofeedback, a technique that uses real-time feedback from electroencephalography (EEG) or functional magnetic resonance imaging (fMRI) to help individuals gain voluntary control over specific neural signals, has shown promise in modulating brain activity patterns implicated in various psychiatric and neurological disorders [13–15].

### (b) Neurofeedback: outline of a promising paradigm for functional neurological disorder

Neurofeedback is a closed-loop neuromodulation paradigm in which individuals receive contingent, real-time feedback derived from ongoing neural activity and are trained to modulate specific neural features through reinforcement-based learning mechanisms [16,17]. A defining characteristic of neurofeedback is the online detection and processing of brain signals, such that feedback is temporally coupled to fluctuations in neural activity [18]. Operationally, a neurofeedback paradigm requires: (i) acquisition of neural signals using EEG, fMRI or functional near-infrared spectroscopy (fNIRS), (ii) real-time signal processing to extract predefined neural parameters (e.g. oscillatory power within a frequency band, regional blood-oxygen-level-dependent activity or measures of functional connectivity), (iii) transformation of these parameters into an interpretable feedback signal, and (iv) reinforcement contingent upon successful modulation of the target signal [19–21]. Interventions lacking temporally contingent neural feedback do not meet the formal criteria for neurofeedback and are more appropriately characterized as non-contingent biofeedback or passive neuromodulation.

From a clinical implementation perspective, neurofeedback is typically delivered across repeated sessions to facilitate gradual acquisition of self-regulatory control through operant conditioning and experience-dependent plasticity [18,22]. Protocols vary according to neural target, signal modality, reinforcement schedule and degree of strategy instruction [23]. Importantly, accumulating evidence suggests that neurofeedback can induce changes not only in regional activation but also in large-scale functional connectivity, supporting its relevance for disorders conceptualized at the network level [24].

Within this framework, neurofeedback offers a particular advantage in the context of FND, insofar as it permits individualized training of neural circuits implicated in symptom generation and self-regulation [25]. Given that FND is increasingly understood as involving dysregulation across sensorimotor, salience and executive control networks, the capacity to tailor training to specific circuit abnormalities aligns with contemporary mechanistic models. Rather than targeting symptoms indirectly, neurofeedback for FND could enable direct engagement with the neural systems hypothesized to underlie disrupted agency, altered motor control and heightened interoceptive or affective processing.

Broadly, neurofeedback applications can be conceptualized within two partially overlapping therapeutic domains. First, motor and sensorimotor modulation paradigms aim to influence oscillatory dynamics or regional activation within sensorimotor cortex, supplementary motor areas (SMAs) or related circuits [26]. Such approaches have been employed in motor rehabilitation and movement disorders, with evidence that sensorimotor rhythm (SMR) training can alter cortical excitability and motor performance [18,27]. Second, cognitively mediated paradigms target executive and limbic circuits—such as dorsolateral prefrontal cortex (DLPFC), anterior cingulate cortex (ACC), insula or amygdala—to enhance top-down regulation, emotional processing and interoceptive control [28]. These approaches are grounded in models of fronto-limbic dysregulation observed across mood and trauma-related disorders [29].

In the context of FND, both domains may be relevant depending on symptom profile and network dysfunction. Functional motor presentations may benefit from direct modulation of sensorimotor circuits implicated in movement initiation and control, whereas stress-sensitive, dissociative or affectively driven symptoms may be more responsive to interventions targeting executive–limbic regulation. Given the heterogeneity of FND, neurofeedback should therefore be conceptualized not as a single uniform intervention but as a family of circuit-targeted training paradigms requiring symptom-informed stratification.

Although previous reviews [1,30] have examined emerging treatments for FND and the broader applications of neurofeedback in neurological and psychiatric disorders, there is currently no integrative perspective that specifically evaluates neurofeedback through the lens of contemporary FND neurobiology, digital health innovation and precision medicine frameworks. The aim of this opinion paper is, therefore, to synthesize emerging mechanistic insights linking dysfunctional brain networks in FND to potential neurofeedback targets, to examine translational evidence from related conditions and to propose a structured conceptual framework for future investigation. Rather than providing a comprehensive systematic review, this article seeks to integrate network-based models of FND with developments in neurofeedback research in order to clarify theoretical plausibility and identify priority areas for empirical study. By considering how neurofeedback might be integrated within established multi-disciplinary FND treatment pathways and existing digital service models, this article outlines its potential role as an adjunctive therapeutic approach grounded in current neuroscientific understanding.

## 2. Theoretical and learning frameworks underpinning neurofeedback

Although neurofeedback is frequently framed as a form of operant conditioning, its mechanisms extend beyond simple stimulus–response reinforcement. Contemporary models conceptualize neurofeedback as engaging reward-based learning, attentional control, error monitoring and experience-dependent neuroplasticity within distributed cortical and subcortical circuits [31,32]. Understanding these mechanisms is essential when considering how different neurofeedback paradigms might be rationally applied in FND.

### (a) Reinforcement learning, reward signalling and motor control

Neurofeedback operates by providing contingent feedback when neural activity approaches a predefined target state. Experimental work demonstrates that reinforcement signals can shape cortical activity patterns via dopaminergic reward pathways, even in the absence of explicit strategy awareness [32,33]. In decoded neurofeedback paradigms, individuals have been shown to modify distributed activation patterns associated with perceptual states without conscious recognition of the target state, illustrating that implicit reinforcement learning can induce measurable neuroplastic change [33].

In FND, where impaired voluntary motor control and altered sense of agency are prominent, reinforcement-based modulation of motor intention circuits (e.g. SMA and premotor cortex) may strengthen adaptive motor representations. If successful motor-related neural states are repeatedly reinforced, this could theoretically stabilize motor network dynamics and reduce aberrant recruitment of salience or limbic systems during movement initiation. Importantly, this mechanism would not rely solely on cognitive reframing but on the gradual reshaping of motor network activity itself.

### (b) Associative learning and intention–action coupling

Beyond classical operant conditioning frameworks, neurofeedback and brain–computer interface (BCI) paradigms may also operate through associative learning mechanisms. In motor rehabilitation contexts, neural signals reflecting movement intention are used to trigger assistive devices—such as robotic actuators or functional electrical stimulation—that facilitate or complete the intended movement. The consistent temporal pairing between endogenous motor preparation signals and congruent sensory or proprioceptive feedback is thought to induce Hebbian-like plasticity within motor circuits [34].

Controlled studies in stroke rehabilitation demonstrate that intention-driven BCI systems can promote clinically meaningful motor recovery, particularly when motor cortical activity is contingently paired with assisted movement execution [35]. More recent work has shown that brain-actuated stimulation paradigms can induce durable motor improvements, suggesting that associative coupling between cortical activation and peripheral movement feedback strengthens functional motor pathways [36]. Reviews of these approaches emphasize that the therapeutic mechanism likely involves restoration of coherent intention–execution coupling through temporally precise contingency learning [37].

This associative framework differs from traditional operant neurofeedback in that the primary therapeutic signal is not simply performance-based feedback but the contingent pairing of neural intention with sensory confirmation of movement. Such mechanisms may have conceptual relevance for motor presentations of FND. If functional weakness, tremor or abnormal movement patterns involve disrupted integration between motor intention and executed action, paradigms that reinforce temporal congruence between preparatory neural activity and successful movement output may help recalibrate intention–action coupling and enhance sense of agency. While direct empirical evidence in FND remains limited, associative neurofeedback models extend the theoretical landscape beyond operant conditioning and warrant consideration in future translational studies.

### (c) Feedback modality, training dose and network plasticity

The modality used to deliver neurofeedback fundamentally shapes both the neural target and the nature of the training signal. EEG-based neurofeedback typically reinforces oscillatory activity within specific frequency bands (e.g. alpha, theta and SMR), thereby influencing large-scale synchronization patterns with high temporal precision [38]. By contrast, fMRI-based neurofeedback provides spatially specific feedback from anatomically defined cortical and subcortical regions, including the amygdala, ACC, insula or DLPFC [39]. More recently, connectivity-based paradigms have emerged, in which feedback reflects the strength of functional coupling between distributed brain regions rather than activity within a single locus [40].

These methodological differences are clinically meaningful. EEG-based approaches may be particularly suited to modulating distributed oscillatory states, whereas fMRI paradigms allow targeted engagement of specific nodes within emotion, salience or motor circuits. Connectivity-based approaches are especially relevant in conditions characterized by disrupted network integration. In FND, where abnormal interactions between salience, executive and motor systems have been proposed to underpin symptom generation, paradigms that directly reinforce functional coupling may offer theoretical advantages. Thus, modality selection should not be viewed as purely technical, but as a decision that reflects the hypothesized circuit-level dysfunction in a given symptom profile.

At the same time, the capacity of any modality to induce meaningful change depends on training intensity and repetition. Neurofeedback effects are unlikely to emerge from single-session exposure. Repeated training is thought to promote synaptic plasticity and induce measurable alterations in resting-state connectivity, with some studies demonstrating sustained neural changes beyond the training environment [41,42]. The consolidation of learned neural regulation appears to depend on cumulative session exposure, spacing of training and opportunities for transfer practice when feedback is withdrawn.

These considerations are particularly relevant in FND, where symptom expression may fluctuate dynamically in response to stress or contextual triggers [43]. Repeated and structured training may help stabilize adaptive network configurations and reduce vulnerability to maladaptive state transitions. However, substantial heterogeneity remains across studies in session number, spacing and reinforcement schedules, limiting direct comparison of outcomes. Identifying optimal ‘dose’ parameters—including frequency, duration and reinforcement structure—for different FND phenotypes represents an important area for future investigation.

### (d) Explicit strategy use, metacognition and predictive updating

Neurofeedback paradigms vary in the degree to which participants are instructed to use explicit cognitive strategies (e.g. motor imagery, emotional recall and attentional focus) versus relying on implicit trial-and-error learning. Evidence suggests that both approaches can produce neural modulation, but individual differences in metacognitive awareness and attentional flexibility may influence success [31].

From a predictive processing perspective, neurofeedback may provide a structured mechanism for updating internal models of bodily control. Real-time feedback reduces uncertainty about internal neural states, potentially recalibrating maladaptive priors that dominate perception and action in functional disorders. In FND, where aberrant top-down expectations about bodily movement or sensation have been proposed [44], contingent neural feedback could theoretically facilitate reweighting of prediction errors and restoration of more adaptive sensorimotor integration.

Explicit strategy-based paradigms may be particularly suitable for patients capable of engaging reflectively with symptom modulation, whereas implicit paradigms may be preferable where conscious effort exacerbates symptoms.

To summarize, neurofeedback is not a single uniform technique, but a flexible approach that can be tailored to specific neural circuits and symptom profiles. By clarifying how dysfunctional brain networks contribute to symptom generation, and how different training parameters shape neural adaptation, a coherent rationale emerges for why neurofeedback may represent a plausible therapeutic approach in FND. The following discussion builds on this foundation to examine how existing clinical and translational evidence supports—and challenges—this potential application.

## 3. Neurofeedback as a promising treatment for functional neurological disorder: targeting brain networks

Neurofeedback leverages the principles of operant conditioning and brain plasticity. By providing individuals with immediate information about their brain activity, it enables them to learn how to self-regulate neural processes. In FND, studies have identified abnormal connectivity and function in circuits involving the prefrontal cortex, insula, ACC and SMA [45–48]. These areas are involved in emotional processing, motor planning and the sense of agency [49–52].

Beyond regional abnormalities, accumulating evidence supports a network-level account of FND pathophysiology. Resting-state and task-based neuroimaging studies have demonstrated altered functional connectivity between limbic regions and motor control areas in individuals with functional movement disorders [53,54]. These findings align with broader models of large-scale brain network organization, particularly the salience network, which integrates interoceptive, emotional and motor-relevant signals [55]. Dysregulation within this network may bias attentional resources towards symptom-related signals while impairing executive modulation of motor output. Such distributed network dysfunction provides a biologically plausible substrate for interventions that aim to stabilize connectivity dynamics rather than target isolated cortical loci.

In particular, FND is thought to involve disrupted communication between cortical regions involved in voluntary motor control and subcortical areas responsible for emotion and arousal [45,56]. This leads to a mismatch between intention and motor execution, often perceived by the person as involuntary [57,58]. Contemporary computational models further conceptualize FND within predictive processing frameworks, in which symptoms arise from abnormal weighting of prior expectations relative to sensory evidence [59]. In this account, dysfunctional motor or perceptual experiences may emerge when top-down predictions dominate bottom-up input, leading to actions that are experienced as involuntary despite intact peripheral systems [54,60]. Clinical theorists have argued that such disturbances in agency and self-monitoring are central to functional symptoms [61]. Within this framework, neurofeedback may serve as a mechanism for recalibrating predictive hierarchies by strengthening the coupling between intentional effort, sensory feedback and measurable neural change, thereby restoring coherence between intention and action.

Neurofeedback may correct these mismatches by reinforcing healthier neural patterns that support accurate self-monitoring and volitional control. Additionally, neurofeedback engages the brain's inherent plasticity by rewarding small, incremental changes in neural activity. Through repeated training, individuals can re-establish functional connectivity in disrupted networks and develop greater control over emotional and motor responses [38,62]. This process has the potential not only to address the physiological underpinnings of FND symptoms but also to enhance psychological constructs such as self-efficacy and perceived control, which are crucial for long-term recovery in FND [63,64].

From a neurophysiological perspective, neurofeedback is understood as a form of closed-loop neuromodulation that leverages reinforcement learning to induce plastic changes in targeted networks [16]. Evidence across psychiatric and neurological conditions suggests that successful neurofeedback training can alter intrinsic connectivity patterns and modulate large-scale network dynamics rather than merely producing transient regional activation changes. Importantly, these neural shifts may coincide with enhanced self-efficacy and perceived control, which are recognized as key therapeutic factors in functional disorders [64,65]. By combining measurable neural modulation with experiential reinforcement, neurofeedback may uniquely bridge biological and psychological mechanisms of change.

For example, targeting increased activation in the DLPFC may enhance executive control over motor function, while downregulating hyperactive amygdala activity could reduce emotional reactivity and stress-induced symptom exacerbation [66–68]. These adjustments align well with the multi-dimensional nature of FND, in which cognitive, emotional and motor domains interact dynamically.

Importantly, neurofeedback protocols can be tailored to the individual, allowing clinicians and users to target specific symptoms or network dysfunctions [69,70]. This personalization increases the relevance and effectiveness of the intervention, particularly in a heterogeneous disorder such as FND.

Taken together, converging evidence from network neuroscience, computational psychiatry and neurofeedback research provides a coherent mechanistic rationale for exploring neurofeedback in FND. Altered connectivity between salience, executive and motor networks has been repeatedly observed in FND and related functional movement symptom presentations [71,72]. Contemporary predictive processing frameworks further posit that aberrant top-down expectations can outweigh bottom-up sensory input, leading to involuntary motor and perceptual experiences despite intact peripheral systems [60].

Neurofeedback, as a closed-loop intervention capable of engaging and modifying large-scale network dynamics [73], represents a theoretically grounded approach that warrants translational investigation. To address the heterogeneity of FND presentations, the following section systematically reviews translational evidence according to key neural circuit domains implicated in symptom generation.

## 4. Clinical applications of neurofeedback for functional neurological disorder

Building on the mechanistic framework outlined above, this section organizes translational evidence according to the neural circuits most consistently implicated in FND: (i) sensorimotor networks, (ii) limbic–salience circuitry, and (iii) executive control systems. Although randomized controlled trials conducted specifically in FND populations remain limited, studies targeting these networks in related conditions provide important insight into the therapeutic plausibility of neurofeedback for FND.

### (a) Sensorimotor network modulation

Disruptions in motor planning and execution networks, including the SMA and sensorimotor cortex, are central to many functional motor presentations. Neurofeedback targeting these systems, therefore, represents a direct translational strategy. EEG-based SMR neurofeedback has demonstrated clinical benefit in chronic pain and affective symptoms. For example, SMR neurofeedback led to faster improvements in pain, depression and anxiety in fibromyalgia compared with escitalopram [74]. Given the overlap between altered sensory processing, affective modulation and motor symptom expression in FND, these findings suggest plausible applicability to functional motor and somatic symptoms.

More directly relevant to motor control, fMRI-based neurofeedback targeting the SMA has demonstrated that participants can learn to voluntarily modulate activity in this region [75]. The SMA plays a key role in voluntary movement initiation and motor intention. The capacity to influence this region through closed-loop training provides proof-of-concept that dysfunctional motor network activity may be amenable to modulation. Recent work examining motor network connectivity in functional movement disorders further supports this approach [76]. Together, these findings suggest that sensorimotor circuits may represent a rational therapeutic target.

### (b) Limbic–salience circuitry

As discussed in §2, limbic and salience networks—including the ACC, insula and amygdala—are pivotal in integrating emotion, arousal and motor output, and dysregulation within these systems may contribute to stress-sensitive symptom exacerbation and altered interoceptive processing [77]. fMRI neurofeedback targeting the ACC has demonstrated improvements in emotional regulation [28]. Given the ACC's role in salience attribution and emotion–action integration, modulation of this region may be particularly relevant for FND presentations characterized by affective instability.

Similarly, neurofeedback-mediated downregulation of insular activity has been shown to reduce hyperarousal states [67]. The insula's central role in interoceptive awareness and salience detection makes it a plausible target for addressing altered bodily perception in FND. In post-traumatic stress disorder (PTSD), for example, amygdala neurofeedback enhanced top-down regulatory control and reduced dissociative symptoms [78]. Although PTSD and FND are distinct conditions, both involve heightened emotional reactivity and disturbances in agency. Modulation of limbic responses through feedback, therefore, offers a potential pathway for reducing stress-triggered symptom amplification in FND.

Emerging neuroimaging meta-analyses also confirm consistent salience network dysregulation across functional neurological presentations [79], further strengthening the translational rationale for targeting these circuits.

### (c) Executive control networks

Executive control systems, particularly involving the DLPFC, are critical for top-down regulation of emotion and motor planning [80], and reduced prefrontal modulation of limbic and motor circuits has been implicated in impaired agency and symptom persistence in FND [81]. Frontal theta modulation through EEG neurofeedback has improved attention and executive functioning in attention-deficit hyperactivity disorder [82]. In depression, augmenting psychotherapy with DLPFC-targeted neurofeedback enhanced clinical improvement [83].

Additionally, fNIRS-based neurofeedback training of the DLPFC has demonstrated improved emotion regulation [84]. Strengthening executive control over limbic and motor systems may therefore address disrupted top-down regulation mechanisms implicated in FND pathophysiology.

Collectively, these studies illustrate the capacity of neurofeedback to enhance executive regulation and voluntary control processes. Although direct trials in FND are required, the alignment between executive network modulation and proposed FND mechanisms provides a coherent translational bridge.

### (d) Cross-condition evidence and shared network mechanisms

Beyond individual circuit targets, broader evidence from chronic pain and psychiatric conditions supports the capacity of neurofeedback to modify large-scale brain networks. A meta-analysis conducted by our group demonstrated reductions in chronic pain following neurofeedback training [85]. Importantly, chronic pain is increasingly conceptualized as a disorder of network dysfunction involving the salience network, default mode network and sensorimotor circuits rather than solely peripheral nociceptive processes [86,87]. Given the documented overlap between pain processing, salience attribution, affective regulation and interoceptive awareness, these findings may be particularly relevant to FND populations presenting with prominent somatic or sensory symptoms.

Similarly, as previously discussed, neurofeedback applications in PTSD have demonstrated improvements in emotional regulation, autonomic stability and symptom reduction [78,88]. Neurofeedback studies targeting amygdala, insula or prefrontal regions have shown that these circuits can be voluntarily modulated through closed-loop training, with corresponding improvements in symptom severity. Although PTSD and FND are diagnostically distinct, both conditions exhibit heightened emotional reactivity, altered interoceptive processing and disturbances in agency, suggesting shared network-level vulnerabilities.

Broader systematic reviews further report neurofeedback efficacy across anxiety and mood disorders [89]. Depression and anxiety are associated with altered connectivity within fronto-limbic and salience networks [29], circuits that overlap substantially with those implicated in FND pathophysiology. Importantly, neurofeedback interventions in these populations have demonstrated not only symptom improvement but also measurable changes in functional connectivity and oscillatory activity, indicating network-level plasticity rather than purely behavioural adaptation [16,22].

These cross-condition findings support three translational inferences relevant to FND. First, large-scale neural networks implicated in FND are demonstrably modifiable through feedback-based training. Second, improvements in affective and somatic symptoms in related conditions suggest that modulation of salience, executive and sensorimotor systems may generalize across diagnostic boundaries where shared mechanisms are present. Third, neurofeedback appears capable of enhancing self-regulation and top-down control—processes central to contemporary predictive and network-based models of FND.

Although these conditions are not identical to FND, they share mechanistic features including disrupted emotion–motor integration, altered salience processing, impaired executive modulation and heightened interoceptive sensitivity. Importantly, the convergent evidence across pain, trauma-related disorders and affective conditions demonstrates that individuals can learn to modulate neural activity within precisely those networks implicated in FND. This convergence reinforces the biological plausibility of circuit-targeted neurofeedback interventions while underscoring the need for FND-specific randomized controlled trials to establish condition-specific efficacy.

## 5. Methodological characteristics of neurofeedback studies and considerations for functional neurological disorder

The direct neurofeedback literature in patients with diagnosed FND remains limited, and no large-scale randomized neurofeedback trials in FND have yet been published. Consequently, methodological characterization must draw on small pilot studies in functional movement presentations and on adjacent motor rehabilitation paradigms targeting similar neural circuits [90]. Across these studies, substantial heterogeneity is evident in the neural signals captured, feedback modalities employed and training dose applied.

In EEG-based approaches applied to functional motor presentations, spectral power within sensorimotor frequency bands (e.g. mu and beta rhythms) recorded over motor cortices has typically served as the feedback signal. Visual feedback—often in the form of moving bars, cursors or task-based graphical displays—has been most commonly used to represent modulation of oscillatory activity. These paradigms parallel established BCI motor rehabilitation frameworks, in which modulation of motor cortical rhythms is contingently paired with visual or device-mediated feedback to promote re-engagement of motor networks [91].

In related motor rehabilitation populations, intention-driven paradigms have demonstrated that pairing motor cortical activity with assisted movement execution can induce clinically meaningful recovery. For example, Ramos-Murguialday *et al.* [35] showed that EEG-based BCI training combined with contingent sensory feedback improved upper-limb function after stroke. Similarly, Biasiucci *et al.* [92] demonstrated that brain-actuated functional electrical stimulation strengthened motor pathways and resulted in sustained motor gains. Reviews of associative motor neurofeedback further emphasize that temporally precise coupling between cortical activation and peripheral movement may promote Hebbian plasticity within motor circuits [37].

While these studies are not specific to FND, they illustrate key methodological principles relevant to its motor presentations: namely, the importance of selecting a neural signal that reliably reflects motor intention and pairing it with congruent feedback capable of reinforcing intention–action coupling. Training dose has varied widely across these related studies. Session numbers range from short multi-session courses to more intensive multi-week protocols, with variability in session length and spacing. Few studies incorporate systematic transfer trials or long-term follow-up, limiting understanding of durability and generalization [27]. In the context of FND—where symptom expression may fluctuate dynamically—clear reporting of session frequency, duration and reinforcement contingencies will be essential for reproducibility and interpretation.

Given the scarcity and heterogeneity of neurofeedback work in FND itself, definitive protocol standardization is premature. However, several pragmatic principles may guide future development:

1. Explicit neural target justification based on established FND pathophysiology.
2. Modality–target alignment, ensuring that the imaging technique reliably captures the spatial scale of the intended signal.
3. Transparent reporting of signal type, feedback modality, reinforcement schedule, session number and spacing.
4. Inclusion of transfer and follow-up assessments to evaluate generalization and persistence.

To summarize, the heterogeneity observed across existing neurofeedback applications—both within FND and in related motor and affective conditions—highlights the early developmental stage of the field. While conceptual alignment between neural targets and symptom profiles is increasingly feasible, methodological consistency remains limited. Establishing clearer reporting standards, refining modality–target matching and systematically evaluating training dose and transfer effects will be essential steps towards building a robust evidence base. These considerations naturally inform the priorities for future investigation, including the design of mechanistically grounded trials, optimization of protocol parameters and integration within multi-disciplinary FND care pathways.

## 6. Future directions for neurofeedback in functional neurological disorder

### (a) Standardization of protocols across studies to enable replication

A critical future step is the development of standardized neurofeedback protocols for FND. To date, neurofeedback studies have varied widely in their EEG targets, session lengths, feedback modalities and outcome measures, making it difficult to compare results or replicate findings. For example, even in well-established applications like slow-cortical-potential training, a systematic review [23] found that protocol details (e.g. number of trials, trial length and artefact handling) differed considerably between studies. Such heterogeneity in methodology undermines scientific rigour and reproducibility. Moving forward, consensus on core training parameters and outcomes would be highly beneficial. In this regard, a recent review conducted by our team on FND neuroimaging [90] noted that inconsistent outcome measures and variable protocols prevented meta-analysis, underscoring the need for uniform methods in future trials. By adopting common frameworks (e.g. standard EEG frequency bands to target, consistent symptom rating scales and agreed criteria for ‘training success’), investigators can better replicate each other's work and build cumulative evidence. Ultimately, protocol standardization has the potential to enable more reliable cross-study comparisons and clarify whether observed clinical improvements are attributable to neurofeedback itself or to idiosyncratic study designs [93]. This concerted effort towards establishing uniform protocols is expected to enhance the reliability of neurofeedback for FND and accelerate its adoption in clinical practice.

### (b) Identification of biomarkers for patient selection and treatment response

To enhance clinical impact, future research should focus on biomarkers that predict which individuals with FND will benefit from neurofeedback and track their responses. FND is a heterogeneous disorder with varying neurobiological profiles; thus, not all patients may respond equally to the same training. Brain-based biomarkers—for instance, specific EEG patterns or neuroimaging findings—could guide patient selection by identifying individuals with the dysfunctional networks that neurofeedback can target [81,94]. In this regard, Perez *et al.* [95] highlighted that on the agenda of ‘FND priorities for future research and practice’, there should be a specific emphasis on elucidating the neurocircuitry of FND, which will aid in the identification of biomarkers to guide treatment selection and prognosis. In practice, this might involve using quantitative EEG (qEEG) or fMRI to screen for abnormalities (e.g. excess frontal theta or limbic hyperconnectivity) that neurofeedback protocols can be designed to modify. Importantly, biomarkers may also indicate treatment responsiveness in FND [96], making them a pivotal factor to focus on, to enhance the uses and applications of neurofeedback for FND.

Moreover, emerging evidence across neuropsychiatric conditions shows that a patient's initial EEG profile in the targeted frequency band often correlates with their ability to learn self-regulation and symptom improvement [97]. Going forward, FND neurofeedback studies should integrate biomarkers into trial design—for instance, by using EEG/fMRI metrics to stratify participants or by adapting protocols in real time to each person's neurophysiology. Biomarker-driven personalization will ensure that neurofeedback is delivered to those with FND most likely to benefit and provide objective indicators of when the training is affecting meaningful neural change.

### (c) Combination therapies integrating neurofeedback with cognitive-behavioural therapy, physiotherapy and mindfulness

Multimodal treatment approaches are poised to enhance outcomes in FND by combining neurofeedback with established therapies, such as CBT, physiotherapy and mindfulness-based interventions. There is a plethora of other conditions for which combining neurofeedback with talk therapy has yielded durable benefits. For example, a recent randomised controlled trial (RCT) focusing on  depression symptoms demonstrated that augmenting standard CBT with a brief course of real-time fMRI-based neurofeedback (training patients to upregulate amygdala activity in response to positive cues) led to greater and more sustained symptom improvement than CBT alone [83]. These findings illustrate the potential synergy between neurofeedback and cognitive-behavioural techniques: neurofeedback directly modifies dysfunctional brain patterns, while CBT reinforces new, healthier thought and behaviour patterns, each potentiating the other's effects.

In the context of FND, combination treatments are already showing promise. For motor FND (functional movement disorders), standard care often involves physiotherapy to retrain movement and CBT to address maladaptive beliefs. A recently published RCT found that a multi-disciplinary programme of physiotherapy plus CBT produced greater improvements than a control intervention in patients with functional movement disorders, improving their physical function and quality of life. Integrating neurofeedback into such programmes could further strengthen outcomes. For example, during physiotherapy sessions for functional limb weakness, EEG neurofeedback could be used to reinforce the person's recruitment of motor networks or to down-regulate hyperactive limbic circuits that interfere with movement. Mindfulness-based strategies are another natural fit. Mindfulness meditation and neurofeedback both aim to improve self-regulation of attention and arousal [98,99]. Studies suggest that these techniques can be mutually reinforcing; for instance, neurofeedback can provide real-time feedback on brain states during mindfulness practice, potentially accelerating the acquisition of meditative self-control [98–100].

In light of this, future treatment models for FND will likely be multi-modal and could incorporate neurofeedback as one component alongside psychotherapy, physical rehabilitation and mindfulness training. Such holistic interventions should aim to tackle the disorder on multiple levels (brain, mind and behaviour), maximizing the chances of meaningful recovery [101,102]. Combination approaches also align with patient-centred care, as they can be tailored to individual needs and delivered by interdisciplinary teams (e.g. neurologists, physiotherapists and psychologists working together) [103–105].

### (d) Development of portable electroencephalography systems for home-based training

Advances in technology are opening the door for home-based neurofeedback using portable EEG devices, which is a crucial future direction to increase treatment accessibility for neurofeedback in FND. Traditional neurofeedback requires frequent clinic visits to sit at a controlled EEG training station, which can be burdensome for patients with limited mobility or resources [106]. Now, lightweight wireless EEG headsets and smartphone apps allow patients to train their brain activity from home under remote supervision [107]. Preliminary studies indicate that home neurofeedback is not only feasible but effective and well-tolerated [108–110]. In a recent proof-of-concept trial, patients with chronic pain were provided with a portable EEG neurofeedback device (‘Axon’) to use at home, completing 32–48 training sessions over eight weeks. In total, 69% of participants reported pain relief alongside improvements in sleep quality and mood, with no serious adverse events noted [108]. This suggests that extended neurofeedback training can be delivered in a home setting with positive outcomes, which could readily translate to FND populations (many of whom also have chronic pain or fatigue as comorbid issues).

The convenience of home-based training may be particularly beneficial for FND use cases, where consistent practice and generalization of self-regulation skills to everyday life are critical. Portable systems enable higher dose and frequency of neurofeedback (daily or multiple times per week) than would typically be possible in clinic, potentially enhancing learning and neuroplasticity. Studies on wearable neurofeedback devices show that they can successfully train brain rhythms; for example, a tiny wireless headset for alpha-wave neurofeedback was shown to significantly increase users’ alpha power and improve their memory performance compared to a sham condition [111]. Such findings validate that lower-cost, mobile technology can deliver neurofeedback training effects.

Moving forward, we expect to see purpose-built home neurofeedback programmes for FND. Importantly, home neurofeedback would ideally be guided by clinicians through telemedicine: therapists could review uploaded EEG session data, monitor compliance and adjust protocols remotely. This model aligns with the broader trend in digital health to empower patients with at-home therapeutic tools while maintaining professional oversight [112,113]. Overall, developing robust portable EEG systems and validating home-based neurofeedback for FND could greatly expand access to this therapy, making long-term self-regulation training practical and scalable beyond specialized clinics [108].

### (e) Use of machine learning to personalize feedback and optimize training trajectories

The application of machine learning (ML) and artificial intelligence in neurofeedback offers exciting possibilities to tailor training to each patient and to optimize learning trajectories in real time. Current neurofeedback protocols often use one-size-fits-all thresholds and heuristics, but individuals differ in their baseline brain activity and how quickly they learn control. ML could revolutionize the field by enabling adaptive, data-driven neurofeedback. A major insight from recent research is that a lack of personalization may contribute to mixed results—in one review [69], investigators noted that using standardized protocols not adjusted to individual neurophysiology could partly explain why some controlled trials found limited neurofeedback benefits.

A future ML-based approach could involve algorithms that continuously analyse the user's EEG performance and dynamically adjust feedback parameters to keep the training optimally challenging and engaging. For example, reinforcement learning algorithms can be employed to set the feedback threshold at an optimized level (neither too easy nor too hard) based on the participant's moment-to-moment performance. Experimental work supports this strategy: in a study with healthy participants [114], a closed-loop adaptive difficulty system that raised or lowered the EEG threshold according to the person's self-rated effort led to improved learning of brain self-regulation compared to a static or random threshold approach. Participants in the adaptive condition achieved higher classification accuracy in hitting the EEG target and reported a more optimal effort level, illustrating that an ML-driven adaptation can accelerate reinforcement learning of brain activity patterns.

ML can also personalize neurofeedback by identifying the most relevant neural features for each patient. Rather than training to target a generic EEG band for all, algorithms might analyse a patient's high-dimensional EEG or fMRI data to pinpoint abnormal network connections or oscillations unique to that individual and then focus the feedback on those features [115,116]. For instance, a recent study [117] utilized an extreme gradient boosting model on pretraining EEG data to predict the response of children with ADHD to neurofeedback, achieving approximately 99% accuracy in classifying responders versus non-responders based on specific EEG frequency patterns in frontal regions. Although this was a small study, it showcases how ML can identify biomarkers of response and thus guide personalized protocol selection. In FND, similar approaches could be used to identify which neural signatures (such as heightened limbic activity or impaired sensorimotor connectivity) are present in an individual and tailor the neurofeedback to address these.

ML algorithms could also continuously optimize training trajectories by learning from prior sessions. For example, if progress stalls, the system might automatically adjust the reward paradigm or switch to training a different network that analysis suggests is more pertinent. Over time, an intelligent neurofeedback platform could ‘learn’ the ideal progression of difficulty and focus for each patient, much like personalized tutors in education. Embracing these AI-driven techniques will require interdisciplinary collaboration (neuroscientists, data scientists and engineers) and careful validation in clinical trials and regulatory considerations on the use of ML in a medical device [118–120]. However, the pay-off would be neurofeedback that is more efficient and effective, adapting to the individual brain in the same way modern precision medicine adapts to the individual body. This could reduce the proportion of FND patients who are ‘non-responders’ by ensuring that everyone gets a training programme optimized for their unique neural profile [69]. Ultimately, ML integration stands to transform neurofeedback from a static intervention into a flexible, evolving learning system that maximizes neuroplastic change for each person.

### (f) Challenges and limitations

Despite the promise of neurofeedback as a brain‑based intervention for FND, several challenges must be addressed before it can be routinely used in community settings. First, placebo responses in FND are often substantial, meaning that sham‑controlled trials with adequate blinding and longer follow‑up are essential to determine genuine treatment effects. Moreover, the current evidence base remains limited. This article has presented a narrative and translational overview rather than a systematic review, and no formal search strategy or study selection protocol was applied. Given the relatively small number of neurofeedback trials conducted specifically in FND populations, we included studies selected for their mechanistic relevance to neural circuits implicated in FND. While this approach allows conceptual integration across related conditions, it does not constitute a comprehensive synthesis and may be subject to selection bias. As the field evolves, systematic reviews and meta-analytic evaluation will be necessary to establish the strength and consistency of treatment effects.

Second, there is marked heterogeneity in neurofeedback protocols. Even so‑called ‘standard’ slow‑cortical‑potential paradigms differ in the number of sessions, trial length, use of transfer trials and outcome metrics [121]. Without consensus on core training parameters and common symptom‑rating scales, results remain difficult to compare across studies. Access to advanced imaging modalities such as real‑time fMRI is also limited to a handful of research centres, and there is uncertainty about how long benefits persist and how easily individuals learn to self‑regulate their brain activity. These issues highlight the need for pragmatic trials that incorporate long‑term follow‑up, evaluate the durability of effects and report on the learning curve.

To build a robust evidence base, future work should focus on standardization, personalization and accessibility. Methodological consensus is needed on the brain targets to train (e.g. SMR and DLPFC), the duration and intensity of training and the behavioural outcome measures used. Such standardization would facilitate reproducibility and meta‑analysis, and clarify whether observed benefits are attributable to neurofeedback itself or to idiosyncratic study designs. At the same time, patient‑level biomarkers should be incorporated into trial design. qEEG or fMRI markers may identify subgroups of FND with disrupted networks amenable to neurofeedback, improving the selection of responders and tailoring protocols. Given that many FND patients require multimodal care [2,122], integrating neurofeedback with physiotherapy, CBT and mindfulness training may yield additive or synergistic benefits. These hybrid models should be evaluated in interdisciplinary care pathways.

Enhancing accessibility is another priority. Most neurofeedback programmes require frequent clinic visits, which can be burdensome for patients with mobility limitations or limited resources [4,123]. Low‑cost wearable EEG devices now make it feasible to record brain activity remotely; such wearables are portable, battery‑powered and inexpensive, and they offer the prospect of frequent, longitudinal home‑based training [124,125]. Preliminary studies in chronic pain and other conditions have shown that home‑based neurofeedback can be delivered safely and effectively under remote supervision (e.g. using smartphone apps and cloud‑based data sharing) [110,126,127]. Future trials should develop and test home‑based neurofeedback programmes specifically for FND, coupled with clinician monitoring and telehealth support. Such developments align closely with the priorities of the NHS Long Term Plan [128], which emphasizes the expansion of digitally enabled care, community-based management of long-term conditions and reduction of health inequalities through improved access to specialist services. Embedding neurofeedback within remote monitoring and integrated care pathways could therefore support NHS objectives of reducing hospital attendance, enhancing patient self-management and shifting care from hospital-centric to community-based models. A parallel effort should evaluate user experience and adherence to ensure that training protocols are acceptable, especially for individuals with fatigue or cognitive difficulties.

Additionally, ML tools offer exciting opportunities to refine neurofeedback, but also introduce new challenges. ML systems can process multimodal data (speech, behaviour and physiological signals) to provide unprecedented levels of personalization, helping clinicians tailor interventions to individual neurophysiology and to predict treatment response [129]. Adaptive algorithms could adjust reward thresholds in real time to optimize learning, while predictive models might determine when to switch targets or intensify training. However, these benefits depend on the quality of the data used; biased or incomplete datasets can produce inaccurate predictions and undermine trust [130]. Moreover, ML-driven tools should augment rather than replace human therapists; clinicians must remain involved to interpret data, safeguard patient autonomy and address ethical issues such as data privacy and the digital divide. Strong regulatory compliance with the use of ML in medical devices will also be required.

Ensuring equitable access will require investment in digital infrastructure, user training and careful consideration of cultural and socioeconomic barriers [131,132]. These factors should be a priority, in tandem with further research into the underlying effectiveness of neurofeedback approaches in FND, rather than a topic to be tackled later.

Lastly, but no less importantly, a further practical challenge concerns the alignment of neurofeedback paradigms with established principles of FND rehabilitation. Contemporary physiotherapy approaches in functional movement disorders emphasize individualized, goal-directed treatment plans that prioritize restoration of functional movement over symptom monitoring, often developed collaboratively with the patient [6,133]. Outcomes are frequently optimized when interventions are framed around meaningful, activity-based goals rather than solely focusing on abnormal movement patterns.

Translating these principles to neurofeedback raises important considerations. While many neurofeedback protocols are standardized around predefined neural targets, FND presentations are heterogeneous and may benefit from greater individual tailoring. Involving individuals with FND in defining functional priorities—such as gait stability, reduction of tremor or improved stress tolerance—may enhance engagement and therapeutic relevance. Broader rehabilitation research suggests that collaborative goal-setting and shared decision-making are associated with improved adherence and treatment outcomes across chronic neurological conditions [134].

However, empirical evidence examining patient co-design or goal-based personalization in neurofeedback for FND is currently lacking. Determining how best to balance standardization with individual tailoring represents an important translational challenge. Future research should examine whether integrating patient-defined functional goals into neurofeedback paradigms improves engagement, perceived agency and clinically meaningful outcomes in this population.

## 7. Conclusive remarks

Neurofeedback represents a biologically informed and technologically evolving intervention that may have relevance for FND. Advances in wearable EEG systems, telehealth platforms and adaptive digital tools have increased the feasibility of remote and community-based delivery. These developments are consistent with the ambitions of the NHS Long Term Plan [128], which prioritizes digitally enabled care, strengthened community services and improved access to specialist expertise for individuals with long-term conditions.

However, the translation of neurofeedback into routine NHS pathways will require careful empirical validation and service-level evaluation. Robust sham-controlled trials, protocol standardization, biomarker-informed stratification and long-term follow-up are needed to clarify clinical efficacy and durability. Implementation research will also be essential to determine feasibility within integrated care systems, assess cost-effectiveness and ensure equitable access across diverse populations. If these scientific, practical and ethical considerations are addressed, neurofeedback may emerge as a complementary adjunct within multi-disciplinary FND care, aligned with broader efforts to modernize and personalize neurological services.

## Data Availability

This article has no additional data.

## Theme

One contribution of 12 to a discussion meeting issue ‘Digital healthcare for the management of functional neurological disorders’.
